# Sexual health status of women who have regular sexual relations with men who have sex with men in mainland China

**DOI:** 10.1186/s12889-017-4096-z

**Published:** 2017-02-06

**Authors:** Xiufang Li, Beichuan Zhang, Juan Wang, Yang Li, Xianhong Li, Peiheng Yu, Minghua Liu, Xinqiao Liu

**Affiliations:** 1 Department of Sex Health Center, Affiliated Hospital of Qingdao University, 16 Jiangsu Street, Qingdao, Shandong 266003 China; 2 Department of Dermatology, Jiaozhou Central Hospital of Qingdao, 29 Xuzhou Street, Jiaozhou, Shandong 266300 China; 3 Department of Dermatology, National Center for STD Control, Chinese Academy of Medical Sciences and Peking Union Medical College, Nanjing, Jiangsu 210042 China; 4 School of Law Central South University, Xiang Ya Nursing School of Central South University, Changsha, Hunan 410013 China; 5 Research Projects of Gay Men’s Wives, Qingdao, Shandong 266012 China; 6 Centers for Disease Control and Prevention of Qingdao, Qingdao, Shandong 266033 China

**Keywords:** Women, Regular sex partner, Men who have sex with men, Sexual health status, Mainland China

## Abstract

**Background:**

Men who have sex with men (MSM) are a high-risk group for sexually transmitted diseases (STDs) and human immunodeficiency virus (HIV) infection. In China, the vast majority of MSM feel forced to marry or plan to marry women, according to traditional Chinese culture. Women who have regular sexual relations with MSM, called *tongqi* in mainland China, live with a high risk of STDs or HIV infection, but these risks are often ignored. Our investigation of this group of the women is a preliminary study that aims to understand the sexual health problems of *tongqi* and related factors.

**Methods:**

This study relied on website mobilization and was funded by *tongqi*. Participants were limited to women who had sex with MSM to whom they were married (in-GWs), whom they had divorced (ex-GWs), or with whom they were friends (GGFs). The data were collected using questionnaire software.

**Results:**

A total 144 valid surveys were returned from 100 in-GWs, 33 ex-GWs, and 11 GGFs. Average respondent age was 32.8 ± 6.4 years (range 22 to 58 years). Among in-GWs and ex-GWs, over 95% learned that their husbands were MSM after marriage. More than half of respondents had had sex before marriage, and one-third of those women had sex partners other than their husbands. In addition, 35.3% of *tongqi* had STDs symptoms. About 50% participants had had oral sex with sex partners of MSM and 10% had had passive anal sex, with low condom use during both oral (9.7%) and anal sex (23.1%). Most *tongqi* had misunderstandings about STDs and HIV and less than 30% had undergone HIV screening. Among participants tested, 5.6% were HIV positive. A total 93.5% of respondents believed that laws should be established to protect the sexual rights of women.

**Conclusions:**

Women who have regular sexual relations with MSM face adverse sexual health issues and are susceptible to STDs and HIV infection. Measures must be taken to protect the rights and interests of *tongqi* in mainland China.

**Electronic supplementary material:**

The online version of this article (doi:10.1186/s12889-017-4096-z) contains supplementary material, which is available to authorized users.

## Background

Women who have regular sexual relations with men who have sex with men (MSM) include three subgroups: 1) women who have sex with MSM to whom they are married; 2) those who had sex with MSM to whom they were married but who they later divorced; and 3) women who have regular sexual relations with MSM but who are unmarried (female friends). These women are called *tongqi* in mainland China, and the three subgroups are respectively designated in-GWs, ex-GWs, and GGFs.

Today, there is a more open attitude toward homosexuality in western countries, with some passing legislation to legalize homosexual marriage. In contrast to these countries, homosexual marriage is illegal in China as well as in many other developing eastern countries. As a result, the status of heterosexual women who are married to MSM varies among different countries worldwide. In developed countries such as the United States or Switzerland, some MSM have regular sex with women or marry women with a different sexual orientation. The political, cultural, and economic backgrounds of such countries permit greater individual freedom of choice and legal protection [1, 2]. Few women have regular sex with MSM, and surveys of these women are focused mainly on qualitative psychology [3–5]. According to a 2002 survey of MSM in the United States, married MSM were more likely to have unsafe sex than unmarried MSM, thus exposing their wives to a higher risk of human immunodeficiency virus (HIV) infection. Future strategies for the prevention of acquired immunodeficiency syndrome (AIDS) among such women should be more targeted [6]. A 2005 statistical analysis of a large sample of MSM in India indicated that 41.8% of MSM were married to women [7]. Data from a 2010 survey of 443 MSM in Tanzania who did not use injection drugs showed that 29.1% of them were married and 11.9% were separated, divorced, or widowed [8]. Another survey of MSM with HIV/AIDS carried out in India showed that women whose sex partners were MSM were at risk for HIV/AIDS [9]. Surveys of MSM with HIV/AIDS in some countries reveal a high percentage are married or have had sex with heterosexual women; however, few surveys have focused on these women [7, 10, 11].

Evidence from recent surveys in China has revealed that the marriage status of MSM to women is significantly different from that in Europe and the United States but is similar to that in many developing countries [12, 13]. A 2011 survey of 318 MSM infected with HIV in China demonstrated that 37.7% of them were married and in some cases, their wives had also been infected with HIV, a situation that should be urgently addressed [14].

The reasons why MSM marry heterosexual women include traditional moral, philosophical, and religious constraints (in which MSM consider a wife and family to act as a “shield” against negative social opinion), a desire for family life and children [6], and unawareness of one’s own sexual identity.

It is worth noting that as a very important group closely related to MSM, *tongqi* receive little attention and support, a phenomenon that is contrary to the requirements of the theory of social construction [15]. Based on this theory, we conducted a survey of *tongqi* from 2011 to 2012 that aimed to understand their physical and mental health problems, marital status, and influencing factors.

## Methods

### Procedure

Our survey relied on “*Tongqi Homeland*”, the first web-based grassroots organization for women who have regular sexual relations with MSM in China, established in 2009. This organization provides services to *tongqi* such as psychological counseling, support, and legal advice for divorce. The survey was mainly mobilized by *Tongqi Homeland* volunteers, most of who had been married to MSM. Women who participated in the survey were required to meet three conditions: 1) they had had regular sex with MSM (this included in-GWs, ex-GWs, and GGFs); 2) they had confirmed that their sex partners were not heterosexual and had had sex with other men; and 3) they had registered on the *Tongqi Homeland* website at least 1 month before the survey began.

The survey included three kinds of questions (Additional file 1) for in-GWs, ex-GWs, and GGFs, to set the polygraph. Research assistants provided the women with detailed information about the survey and obtained verbal informed consent. Women consented to participate in the survey anonymously, so records by name were not maintained.

A one-on-one mode of recruitment was used for the survey. Subjects were identified by phone or using the online platform. User keys that could only be used with one computer were automatically generated for all subjects. Survey software (http://www.sojump.com) automatically recorded the reading time and filtered out questionnaires that were submitted in less than 10 min. Links to the questionnaires were not sent over public Internet servers but rather were sent directly to subjects using instant messaging software such as QQ. Surveys were automatically saved and remained active for several hours until completion. User keys were automatically canceled after a questionnaire was completed and submitted. The survey remained active for 3 months (from April to June 2012).

The survey procedure was approved by the Institutional Review Board of the Affiliated Hospital of Medical College, Qingdao University.

### Measures

Information on participants’ demographic characteristics included age, residence (rural, urban), present residence address, educational background, whether the last marriage was the first marriage (yes, no), and whether the participant had children (yes, no).

Information on participants’ sex behaviors included whether they had had sex before marriage, the frequency of their sexual activities, whether participants had had oral or anal sex, whether they had been abused during sexual activity, whether they had sex with men other than their husbands, and the reasons for having sex with other men.

Information on sexually transmitted diseases (STDs) and HIV included four aspects. First, knowledge and attitudes about STDs and AIDS were queried by the following questions: Do a man’s normal-looking genitalia indicate that he has no venereal diseases?, Are some STDs still infectious even though they are completely cured?, Can some STDs cause death?, Do women with syphilis, gonorrhea, and other STDs have no clinical manifestations for a long time?, Can AIDS be transmitted through blood, from mother to child, and by sexual contact?, Is it difficult to detect HIV infection because there are often no symptoms for some time?, Is HIV infection similar to AIDS?, Do people with AIDS feel that life is meaningless?, Is the risk of HIV infection higher after learning that sex partners are MSM?, Should women who are the sex partners of MSM undergo testing for HIV infection?

The second aspect of information about STDs or HIV infection and testing included the prevalence of STD/HIV infection and whether participants had been tested for STDs or HIV. The third involved the use of condoms and whether participants used condoms during vaginal, oral, or anal sex; the frequency of condom use; and whether participants would change their habitual condom use during sex after learning that their sex partners were MSM. Fourth, the topic of abortion was approached by asking whether participants had had an abortion and whether they knew that their sex partners were MSM when deciding to have an abortion.

As for sex education and the right to sex, we asked the following questions: When participants were told that their sex partners are MSM, did they believe that being MSM was a common phenomenon?, After divorcing an MSM, do participants then suspect that all men that they have encountered are MSM?, Can marrying an MSM cause women serious harm?, Should sex outside of marriage be okay when there is no normal sex life in the marriage?, Do MSM who conceal the truth so as to get married seriously harm the rights of women?, Is there a lack of public education about sexuality in the country?, Should there be improvement in education about sexuality?, Should society accept MSM?, Should the rights and interests of MSM be protected so that they do not feel that they must marry women?, Should legal protection of the rights and interests of *tongqi* be instated?

Attitudes toward homosexuality were also investigated through the multiple-choice question, “What do you think about homosexuality?” Response options given were that it is sexual perversion, a mental illness (psychological barrier), a moral issue, or a normal phenomenon.

## Results

### Demographic characteristics

A total 147 surveys were returned, 144 of which were valid. Participants included 100 in-GWs, 33 ex-GWs, and 11 GGFs. The average age of participants was 32.8 ± 6.4 years (median 31 years, range 22–58 years). Among in-GWs and ex-GWs (*n* = 132), the average age at the last marriage was 26.0 ± 3.3 years (median 26 years, range 20–36 years). Most participants came from urban areas (79.2%, 114/144), had recently lived in a first-tier or second-tier city (38.9%, 56/144), and had college-level or higher education (74.3%, 107/144). A total 96.2% (127/132) of *tongqi* were in their first marriage, and more than two-thirds of them had children (71.4%, 95/133) (Table 1).

**Table 1:** Demographic characteristics

Variables	Number	Percentage (%)
1. Age (*N* = 142)		
20–30 years	46	32.4
30–40 years	76	53.5
≥ 40 years	20	14.1
2. Age at the last marriage (*N* = 132)		
20–30 years	115	87.1
30–40 years	17	12.9
3. Residence (*N* = 144)		
Urban	114	79.2
Rural	30	20.8
4. Present residence address (*N* = 144)		
First-tier or second-tier city	56	38.9
Third-tier city	40	27.8
Small city (including the country)	34	23.6
Village or town	14	9.7
5. Educational background (*N* = 144)		
Middle school or lower	12	8.3
High or technical secondary school	25	17.4
College or higher	107	74.3
6. Last marriage was the first marriage (*N* = 132)		
Yes	127	96.2
No	5	3.8
7. Participant has children (*N* = 133)		
Yes	95	71.4
No	38	28.6

### Sex behaviors

Before marriage, more than half (54.9%, 73/133) of *tongqi* had had sex with MSM. Of those women, only 28.6% (20/70) took the initiative for sex, 69.0% (49/71) reported that their partners could successfully perform sexual intercourse, and 95.5% (127/133) learned that their husbands were MSM after marriage. A total of 18.4% (23/125) had sex eight times or more per month; 6.4% reported having had sex eight times or more in the last 6 months (6/94). Among participants, 56.5% (70/124) had oral sex with MSM and 10.9% (13/119) had anal sex. One-tenth of participants were abused during sex intercourse (9.9%, 13/131), and only 25.0% (32/128) felt that they had their sexual needs met. One-fifth of *tongqi* had previously had sex once or twice per week (22.0%, 28/127), but the frequency of sex was significantly reduced (25.0%, 30/120) after learning that their sex partners were MSM. A total of 62.0% (80/129) reported feeling depressed when there was no sexual intercourse. One-third of *tongqi* (32.1%, 42/131) had sex with other men while married. These women reported that the main reasons for engaging in extramarital sex included: 1) to satisfy sexual desire (36.1%, 13/36); 2) to feel more self-confident (30.6%, 11/36); 3) to vent feelings of frustration with their husbands (16.7%, 6/36); and 4) they had had sex outside marriage before learning that their sex partners were MSM (2.8%, 1/36) (Table 2).

**Table 2:** Sex behaviors

Variables	Number	Percentage (%)
1. Had sex before marriage (*N* = 133)		
Yes	73	54.9
No	60	45.1
2. Took the initiative for sex before marriage (*N* = 70)		
Yes	20	28.6
No	50	71.4
3. Sexual intercourse performance (*N* = 71)		
Completed satisfactorily	49	69
Not completed satisfactorily	3	4.2
With the help of *tongqi*	7	9.9
With the help of drugs	1	1.4
With the aid of stimulation such as videos	2	2.8
Uncertain	9	12.7
4. Learned that husband is MSM after marriage (*N* = 133)		
Yes	127	95.5
No	6	4.5
5. Highest frequency of sexual activity (*N* = 125)		
More than 8 times per month	23	18.4
4 to 7 times per month	23	18.4
1 to 3 times per month	32	25.6
Once every 1 to 3 months	12	9.6
Several times in 1 year	35	28
6. Frequency of sexual activity in the last 6 months (*N* = 94)		
More than 8 times per month	8	6.4
4 to 7 times per month	5	5.3
1 to 3 times per month	17	18.1
1 to 5 times in total	27	28.7
No sex	39	41.5
7. Oral sex (*N* = 124)		
*Tongqi* performs for MSM	47	37.9
MSM performs for *tongqi*	7	5.6
Mutual oral sex	16	12.9
No oral sex	54	43.5
8. Anal sex (*N* = 119)		
Yes	13	10.9
No	106	89.1
9. Abused during sex intercourse (*N* = 131)		
Yes	13	9.9
No	118	90.1
10. Attitude of MSM toward sexual desires of spouse (*N* = 128)		
Disinterested	36	28.1
Cooperative	34	26.6
Meets spouse’s needs	32	25
Ridicules and refuses sex	8	6.3
All of the above	18	14.1
11. Sex frequency before learning that sex partner is MSM (*N* = 127)		
1 to 2 times per week	28	22
1 to 3 times per month	47	37
Rarely	52	40.9
12. Sex frequency after learning that sex partner is MSM (*N* = 120)		
Significantly reduced	30	25
Increased	13	10.8
Similar to previously	24	20
Nearly or completely stopped	53	44.2
13. Participant’s attitude when there is no sexual intercourse (*N* = 129)		
Depressed	80	62
Indifferent	25	19.4
Uncertain	24	18.6
14. Sex with other men while married (*N* = 131)		
No	89	68
One partner	27	20.6
2 to 5 partners	13	9.9
More than 5 partners	2	1.5
15. Main reasons for engaging in extramarital sex (*N* = 36)		
Satisfy sexual desires	13	36.1
Feel more self-confident	11	30.6
Vent feelings of frustration with husband	6	16.7
Sex outside marriage before knowing the truth	1	2.8

### STDs and AIDS

#### Knowledge and attitudes about STDs and AIDS

Most *tongqi* had misunderstandings about HIV/AIDS and STDs. They were asked about their attitudes using the following statements related to STDs and HIV: 1) Normal genitalia in men means that they have no venereal diseases. 2) Some STDs are still infectious even though they are completely cured. 3) Some STDs can cause death. 4) Women with syphilis, gonorrhea, and other STDs have no clinical manifestations for a long time. 5) AIDS can be transmitted through blood, from mother to child, or by sexual contact. 6) It is difficult to detect HIV infection because there are no symptoms for some time. 7) HIV infection is similar to AIDS. 8) People with AIDS feel that life is meaningless. 9) After learning that their sex partners are MSM, the risk of HIV infection for women increases. 10) Women who are the sex partners of MSM should undergo HIV testing (Table 3).

**Table 3:** Knowledge and attitudes about STDs and AIDS

	Number	Yes(%)	No(%)	Uncertain(%)
1. Do a man’s normal-looking genitalia indicate that he has no venereal diseases?	132	35.6	27.3	37.1
2. Are some STDs still infectious even though they are completely cured?	130	49.2	12.3	38.5
3. Can some STDs cause death?	133	82.7	3.8	13.5
4. Do women with syphilis, gonorrhea, and other STDs have no clinical manifestations for a long time?	128	33.6	23.4	43.0
5. Can AIDS be transmitted through blood, from mother to child, and by sexual contact?	133	42.1	0	57.9
6. Is it difficult to detect HIV infection because there are often no symptoms for some time?	129	64.3	11.6	24.0
7. Is HIV infection similar to AIDS?	131	6.9	69.5	23.7
8. Do people with AIDS feel that life is meaningless?	134	63.4	26.9	9.7
9. Is the risk of HIV infection higher after learning that sex partners are MSM?	126	70.6	18.3	11.1
10. Should women who are the sex partners of MSM undergo HIV testing?	133	81.2	8.3	10.5

#### STD infection and testing

A total 35.3% (36/102) of in-GWs and ex-GWs had one or several symptoms of STD infection including abnormal vaginal discharge (50.0%; 18 women), burning pain (36.1%; 13 women), abnormal urethral discharge (27.8%; 10 women), pain or ulceration of the vagina or vulva (16.7%; 6 women), anal discharge (5.6%; 2 women), anal ulcers or pain (2.8%; 1 woman), inguinal lymph node swelling (2.8%; 1 woman), or other symptoms (5.6%; 2 women). A total 79.4% (27/34) of participants had received treatment for their symptoms.

A total 15.2% (20/132) of MSM had spread STDs to their sex partners; 78.0% of participants (103/132) had no infections and 6.8% (9/132) were uncertain.

The types of STDs infections reported by participants included condyloma acuminatum 25.0% (5/20), chlamydia 10.0% (2/20), syphilis 5.0% (1/20), gonorrhea 5.0% (1/20), mycoplasma infection 5.0% (1/20), pubic lice 60.0% (12/20), genital herpes 0% (0/120), and others 5.0% (1/20). Nine subjects were infected with a “legally” reported STDs.

After the survey, 27.0% (33/122) of participants said they would undergo testing for STDs, 42.6% (52/122) said they would not, and 30.3% (37/122) were undecided.

#### HIV infection and testing

A total 28.5% (37/130) of subjects had undergone HIV testing with 5.6% (2/36) testing positive and 88.9% (32/36) testing negative; 5.6% (2/36) did not know their test results. A total 19.7% (15/76) of participants who had not undergone HIV testing indicated that they would do so, 53.9% (41/76) said they would not, and 26.3% (20/76) were undecided.

A total 21.3% (26/122) of participants reported that their sex partners had undergone HIV testing, 20.5% (25/122) reported their sex partners had not been tested, and 58.2% (71/122) were uncertain. Four (16.0%) participants reported that their sex partners had tested positive for HIV infection, 19 (76.0%) reported negative test results, and two (8.0%) were uncertain.

After the survey, 24.8% (30/121) of participants reported that they would undergo HIV testing, 46.3% (56/121) said they would not, and 28.8% (35/121) were undecided.

Two ex-GWs were infected with HIV as were their ex-husbands; one in-GW was not infected with HIV, but her husband was HIV-positive; and one GGF had not undergone testing, but her regular sex partner had tested positive for HIV.

#### Condom use

A high proportion of *tongqi* never used condoms during vaginal sex (40.3%, 52/129), oral sex (90.3%, 56/62), or anal sex (76.9%, 10/13). After learning that their sex partners were MSM, the frequency of consistent condom use increased from 22.4% (15/67) to 53.6% (30/56). After the survey, 41.9% (49/117) of participants reported that they would use condoms during every instance of sexual intercourse (Table 4).

**Table 4:** Condom use

Variables	Number	Percentage (%)
1. Condom use during vaginal sex (*N* = 129)		
Yes	77	59.7
No	52	40.3
2. Condom use during oral sex (*N* = 62)		
Yes	6	9.7
No	56	90.3
3. Condom use during anal sex (*N* = 13)		
Yes	3	23.1
No	10	76.9
4. Frequency of condom use before learning sex partner is MSM (*N* = 67)		
Never	10	14.9
Occasionally	19	28.4
Sometimes	11	16.4
Frequently	12	17.9
Consistently	15	22.4
5. Frequency of condom use after learning sex partner is MSM (*N* = 56)		
Never	6	10.7
Occasionally	9	16.1
Sometimes	5	8.9
Frequently	6	10.7
Consistently	30	53.6
6. Frequency of condom use after survey (*N* = 117)		
Frequently	34	29.1
Consistently	49	41.9
Same as before the survey	34	29.1

#### Abortion

A total 71.5% (103/144) of women reported having had an abortion. Of these, 13.6% (14/103) had known their sex partners were MSM (including 11 in-GWs and three ex-GWs).

### Sex education and the right to sex

When they were told that their sex partners were MSM, most *tongqi* believed that the phenomenon of MSM was common (92.6%, 126/136); after divorcing, they suspected that all men they had encountered were MSM (61.3%, 19/31). In addition, most women felt that marrying an MSM is harmful to women (81.5%, 106/130), that sex outside of marriage should be okay if there is no normal sex life in the marriage (60.1%, 83/138), that MSM who concealed the truth to get married seriously harmed the rights of women (91.1%, 123/135), and that public education about sexuality was lacking in China (85.0%, 119/140). As a result, a high percentage of *tongqi* believed that measures should be taken to protect the rights of women and MSM, including improving education about sexuality (92.7%, 127/137), fostering social acceptance of MSM (52.5%, 73/139), safeguarding the rights and interests of MSM so that they do not feel they must marry women (67.6%, 94/139), and enacting legal protection of the rights and interests of *tongqi* (93.5%, 130/139) (Table 5).

**Table 5:** Sex education and the right to sex

	Number	Yes(%)	No(%)	Uncertain(%)
1. When told that their sex partner is MSM, did participant believe that MSM are a common phenomenon?	136	92.6	7.4	0
2. After divorcing an MSM, does participant suspect that all men she has encountered are MSM?	31	61.3	38.7	0
3. Can marrying an MSM cause women serious harm?	130	81.5	8.5	10.0
4. Should sex outside of marriage be okay when there is no normal sex life in the marriage?	138	60.1	10.1	29.7
5. Do MSM who conceal the truth so as to get married seriously harm the rights of women?	135	91.1	2.2	6.7
6. Is there a lack of public education about sexuality in the country?	140	85.0	6.4	8.6
7. Should there be improvement in education about sexuality?	137	92.7	0.7	6.6
8. Should society accept MSM?	139	52.5	14.4	33.1
9. Should the rights and interests of MSM be protected so that they do not feel that they must marry women?	139	67.6	11.5	20.9
10. Should legal protection of the rights and interests of *tongqi* be instated?	139	93.5	1.4	5.0

Finally, 25.7% (36/140) of participants considered homosexuality to be sexual perversion, 34.3% a mental illness (48/140), 8.6% a moral issue (12/140), and 31.4% a normal phenomenon (44/140).

## Discussion

This survey of women whose regular sex partners were MSM represents original exploratory research of sex and reproductive health among *tongqi* in mainland China.

Traditional Chinese culture highly advocates heterosexual monogamous relationships, with procreation considered the main purpose of sex and marriage [16]. Therefore, nearly all MSM feel obligated to marry women. This survey of women who were married to and whose sex partners were MSM confirmed this fact. It has been estimated that more than 90% of MSM marry women or believe that such marriage is inevitable [17]. In many surveys of MSM during recent years in China, social scholars have pointed out that unmarried MSM generally choose traditional marriage regardless of their true wishes [18].

There are some uncertain factors when estimating the number of *tongqi* in China. According to a population sample of the National Bureau of Statistics of China in 2012, the number of men whose first marriage was to a woman was 402.871 million [19]. According to data of Pan and Liu et al. [15, 17], the total number of MSM in China is 7.614–10.515 million (M = 9.065 million). In addition, there are many women who have sex with men but are unmarried; thus, it can be estimated that there are nearly 10 million *tongqi* in mainland China. These women are at high risk of STDs and HIV infection related to sexual contact with MSM. Our survey revealed that the health problems of *tongqi* are often ignored for extended periods by medical and health care professionals.

Sex discrimination (the belief that women are inferior to men) has been a part of traditional Chinese culture since ancient times. This belief has a powerful and widespread influence on Chinese people. The principles of love and marriage are established by the patriarchal authority of the husband, attitudes of male chauvinism (women are expected to show obedience to the father before marriage then obedience to the husband after marriage, the husband controls the wife, and so on), and heterosexism [20]. These often result in stigmatization by sex, placing women at a disadvantage with respect to physical and mental health; their needs, such as an equal right to sex, are usually ignored. Some in the MSM community are firmly opposed to conducting surveys of *tongqi* for fear of discrimination because of their sex orientation [21]. Thus, carrying out relevant surveys is usually met with great resistance.

One recent survey showed that 60% of heterosexual married people who were under 40 years old had sex two or more times a week. In the last year, men who performed oral sex for women during sexual intercourse accounted for 38.8% of those surveyed; that percentage for women who performed oral sex for men was 35.2%. A total 8.5% of respondents had anal sex. The incidence of self-reported STDs among heterosexual people was 2.5% and that among “non-heterosexuals” was 33.6%; the risk of STDs infection among the latter group was 22.6 times that of the former. A total 9.7% of women reported that they had sex with other men while married [15]. This survey revealed that sex behaviors were clearly different for *tongqi* after becoming the sex partners of MSM.

A survey of 557 MSM in Nigeria who had had anal sex revealed that 48.1% had had sex with women in the past 2 months. Among those MSM, 45.1% had had anal sex, 74.0% had not taken protective measures, and 66% had had unprotected vaginal intercourse. Bisexual sex among MSM was related to being married to or living with women [22]. A large systematic review of data collected from the published literature showed that only 10% to 20% of younger MSM got married in China, but more than 50% married at older ages, had lower socioeconomic status, or made contact in certain places [23–25]. Surveys have also revealed that married MSM were more often infected with STDs or HIV than unmarried MSM [26, 27]. According to another survey, less than 50% of MSM with AIDS had told their wives about their disease, and 28.6% of their wives had been infected with HIV [28]. Other surveys have indicated that MSM in China often have STDs. A survey in China of MSM with HIV and syphilis suggested that more than 40% have syphilis [29]. Compared with women sex workers, *tongqi* are more often infected with HIV because of engaging in unprotected sex with MSM or ignorance of their sex partners’ sexual activities with men. This study proved that the level of risk for STD infection among this group of women was high, similar to that of MSM. *Tongqi* are vulnerable to the spread of STDs or HIV from MSM, a situation that has long been ignored. It is worth noting that the knowledge possessed by *tongqi* about these risks is far from being effective to prevent and treat STDs and HIV, with only a few women having undergone testing. In this study, most *tongqi* still had a negative attitude toward STDs or HIV testing, even after having received appropriate information. After learning that their partner was an MSM and having acquired some understanding about the relationship between being an MSM and STDs, some women stated that they would reduce sexual intercourse and increase the use of condoms to prevent sexually transmitted diseases; however, the risks of infection associated with premarital and extramarital sexual behavior remain. Additionally, it is difficult for some in-GWs to divorce their MSM spouses, so they are forced to live with the high risk of STDs for many years.

This survey highlighted that most *tongqi* in our sample were quite positive about sex education. Our study also revealed the important fact that although only a handful of the women surveyed understood that modern science considers same-sex love to be a normal phenomenon within a minority group, most women in the sample were quite positive about sex education owing to their own unique experiences in marriage. A high percentage of the *tongqi* in our study realized that it was important to legally protect the rights of those with a different sex orientation and gender. Some surveys in China have introduced a perception of MSM that is in the interest of sustainable social development and have explained preliminarily the relationship between the AIDS epidemic among MSM and the protection of human rights [30–32]. Today, the lack of awareness about different types of interdependence, interaction, and mutually supportive relationships with respect to sex orientation and gender discrimination can result in the emergence of various human health problems.

“Women and AIDS” by the Joint United Nations Programme on HIV/AIDS (UNAIDS) points out that AIDS prevention among women is often neglected and recommends preventive measures, which are often unable to be controlled by women. This situation could be changed by challenging social ignorance, providing women with support services, creating a safer environment for women, reducing the vulnerability of women through policy reforms, and other similar approaches. Organizations of women and gender in China have reported that gender inequality is the most common type of discrimination and that the intersection between marriage and sexual orientation serves to place women at the greatest disadvantage because multiple social identities must be maintained. These fit with various characteristics of Chinese women whose regular sex partners are MSM. Medical and health care staff in mainland China would benefit from the vigorous promotion of bioethics education on gender and sex equality and protection of the rights and interests of *tongqi*, to improve awareness about the health of this group. With greater openness and awareness about human rights in Chinese society, it is expected that the number of women who marry MSM will decrease, as will the occurrence of associated health problems.

It should be mentioned that our analysis was from a methodological point of view, which has limitations for the survey of a small part of this social group [15]. The participants in our survey were not randomly selected but were drawn from volunteers via a website. Women who were concerned about and knew their husband’s sexual history or who were unhappy in their marriage took part in this survey; thus, the results may not representative of the entire population of *tongqi* in China. Our survey leaves many questions unresolved. It is conceivable that further social changes will emerge, enabling larger sample surveys to be conducted in the future that address the physical and mental health of *tongqi* in China.

## Conclusions

In traditional Chinese culture, procreation is considered to be the main purpose of sex and marriage; therefore, nearly all MSM feel obligated to marry women. Most of the 144 *tongqi* surveyed in the present study did not know that their sex partners were MSM before getting married. One-tenth of participants were abused during sexual intercourse. During sex with their MSM sex partners or with other sex partners, some *tongqi* had had oral sex and anal sex with little or no condom use. Most of the women surveyed had misunderstandings about HIV/AIDS and STDs and some had symptoms of STDs. A few had undergone HIV testing, with 5.6% testing HIV-positive. Most participants felt that there was a great lack of sex education in mainland China and that improvement in sex education was needed. Most participants thought protecting the rights of MSM was also important for the rights of women and that relevant laws should be established to protect women’s sexual rights. Taken together, our analyses indicate that women who have regular sexual relations with MSM are often ignored for long periods; they therefore face adverse sexual health issues and are susceptible to HIV and STDs. Proper measures must be taken to protect the rights and interests of *tongqi*, such as by educating MSM and society as a whole, to improve equality of gender and sex orientation.

## Additional file

Additional file 1: Questionnaire for *Tongqi*. (DOC 77 kb)

## Abbreviations

AIDS: Acquired immunodeficiency syndrome
Ex-GWs: Women who had sex with MSM whom they had divorced
GGFs: Women who had sex with MSM with whom they were friends
HIV: Human immunodeficiency virus
In-GWs: Women who had sex with MSM to whom they were married
MSM: Men who have sex with men
STDs: Sexually transmitted diseases
UNAIDS: Joint United Nations Programme on HIV/AIDS
