# Supplementary material for: Sexual health status of women who have regular sexual relations with men who have sex with men in mainland China
Source: BMC Public Health. 2017 Feb 6;17:168. doi: 10.1186/s12889-017-4096-z (PMC5294694; doi:10.1186/s12889-017-4096-z)
Supplement: Additional file 1: — Questionnaire for Tongqi. (DOC 77 kb) [file 12889_2017_4096_MOESM1_ESM.doc]

**Questionnaire for *Tongqi***

1. **Demographic characteristics**
2. I am ____ years old.
3. I was ____ years old at my last marriage.
4. Residence

Urban Rural

(4) Present residence address

First-tier or second-tier city Third-tier city Small city (including the country) Village or town

(5) Educational background

Middle school or lower High or technical secondary school College or higher

(6) I was in my first marriage when married an MSM.

Yes No

(7) My husband and I have children. Yes No

2. **Sex behaviors**

(1) I have had sex with MSM before marriage. Yes No

(2) If the answer to (1) above is yes, please complete the following:

I took the initiative for sex before marriage. Yes No

(3) If the answer to (1) above is yes, please complete the following:

Describe how sexual intercourse is performed. Completed satisfactorily Not completed satisfactorily With my help With the help of drugs With the aid of stimulation such as a video Uncertain

(4) I learned that my husband was an MSM after marriage. Yes No

(5) Greatest frequency of sexual activity. More 8 times per month 4 to 7 times per month 1 to 3 times per month Once every 1 to 3 months Several times a year

(6) Frequency of sexual activity in the last 6 months. More than 8 times per month 4 to 7 times per month 1 to 3 times per month Total of 1 to 5 times I have not had sex

(7) Describe your experience of oral sex. I perform oral sex for my sexual partner My sexual partner performs oral sex for me My sexual partner and I have mutual oral sex My sexual partner and I do not have oral sex

(8) I have anal sex. Yes No

(9) I am abused during sexual intercourse. Yes No

(10) Describe the attitude of MSM towards spouse’s sexual desires. Disinterested Cooperative Meets their spouse’s needs Ridicules and refuses spouse All of the above

(11) Sex frequency before learning that sex partner was an MSM.

1 to 2 times per week 1 to 3 times per month Rarely

(12) Sex frequency after learning that sex partner was an MSM. Significantly reduced Increased Similar to previously Nearly or completely stopped

(13) My attitude when there is no sexual intercourse. Depressed Indifferent Uncertain

(14) I have had sex with other men while married. No One partner 2 to 5 partners More than 5 partners

(15) My main reason for engaging in extramarital sex.

To satisfy sexual desire To feel more self-confident To vent feelings of frustration with my husband I had sex outside of marriage before learning that my sex partner

was an MSM

3. **STDs and AIDS**

*Knowledge and attitudes about STDs and AIDS*

(1) Normal genitalia in a man means that they have no venereal diseases. Yes No Uncertain

(2) Some STDs are still infectious even though they are completely cured. Yes No Uncertain

(3) Some STDs can cause death. Yes No Uncertain

(4) Women with syphilis, gonorrhea, and other STDs have no clinical manifestations for a long time. Yes No Uncertain

(5) AIDS can be transmitted through blood, from mother to child, or by sexual contact. Yes No Uncertain

(6) It is difficult to detect HIV infection because there are no symptoms for some time. Yes No Uncertain

(7) HIV infection is similar to AIDS. Yes No Uncertain

(8) People with AIDS feel that life is meaningless. Yes No Uncertain

(9) After learning that their sex partners are MSM, the risk of HIV infection for women increases. Yes No Uncertain

(10) Women who are the sex partners of MSM should undergo HIV testing. Yes No Uncertain

*STD infection and testing*

(1) I have one or more symptoms of an STD infection. Yes No

(2) Symptoms that I have include: Abnormal vaginal discharge Burning pain Abnormal urethral discharge Pain or ulceration of the vagina or vulva Anal discharge Anal ulcers or pain Inguinal lymph node swelling Other symptoms

(3) If you have an STD, complete the following:

I have received treatment for STD symptoms. Yes No

(4) My sex partners have spread STDs to me. Yes No Uncertain

(5) The types of STDs that I have been infected with include: Condyloma acuminatum Chlamydia Syphilis Gonorrhea Mycoplasma infection Pubic lice Genital herpes Other

(6) I have been infected with a “legally” reported STD. Yes No

(7) After the survey, I will undergo testing for STDs. Yes No Uncertain

*HIV infection and testing*

(1) I have undergone HIV testing. Yes No

(2) If the answer to (1) above is yes, please complete the following:

My HIV test results were: Positive Negative Uncertain

(3) If you have not undergone HIV testing, please complete the following: I will undergo HIV testing after the survey. Yes No Undecided

(4) My sex partners have undergone HIV testing. Yes No Uncertain

(5) If you know your sex partners have undergone HIV testing, please complete the following:

My sex partners’ HIV test results were: Positive Negative Uncertain

(6) After the survey, I will undergo HIV testing. Yes No Undecided

*Condom use*

(1) My sexual partner and I use a condom during vaginal sex. Yes No

(2) If you have oral sex, please complete the following:

My sexual partner and I use a condom during oral sex. Yes No

(3) If you have anal sex, please complete the following:

My sexual partner and I use a condom during anal sex. Yes No

(4) If you have used a condom, please complete the following:

Frequency of condom use before learning my sex partner is an MSM. Never Occasionally Sometimes Frequently Consistently

(5) If you have used a condom, please complete the following:

Frequency of condom use after learning my sex partner is an MSM. Never Occasionally Sometimes Frequently Consistently

(6) Frequency of condom use after this survey. Frequently Consistently Same as previously

*Abortion*

(1) I have had an abortion. Yes No

(2) If the answer to (1) above is yes, please complete the following:

I have known my sex partner is an MSM. Yes No

**4. Sex education and the right to sex**

(1) When I was told that my sex partner was an MSM, I thought this was a common phenomenon. Yes No Uncertain

(2) After divorcing, I now suspect that all men I have encountered are MSM. Yes No Uncertain

(3) Marrying an MSM is harmful to women. Yes No Uncertain

(4) Sex outside of marriage should be okay if there is no normal sex life in the marriage. Yes No Uncertain

(5) MSM who conceal the truth so as to get married seriously harm the rights of women. Yes No Uncertain

(6) Public education about sexuality is lacking in China. Yes No Uncertain

(7) Education about sexuality should be improved. Yes No Uncertain

(8) Society should accept MSM. Yes No Uncertain

(9) The rights and interests of MSM should be protected so that they do not feel that they must marry women. Yes No Uncertain

(10) Legal protection of the rights and interests of *tongqi* should be instated. Yes No Uncertain

(11) I consider homosexuality to be: Sexual perversion A mental illness A moral issue A normal phenomenon

Note: The content included in our study is only a small part of the entire questionnaire for *tongqi*. Therefore, we have only provided the portion of the questionnaire that is applicable to our study. To obtain a copy of the entire questionnaire, please an e-mail Xiufang Li (lxfqd@126.com) to receive a Chinese version.
